# Exploring social media determinants in fostering pro-environmental behavior: insights from social impact theory and the theory of planned behavior

**DOI:** 10.3389/fpsyg.2024.1445549

**Published:** 2024-07-31

**Authors:** Chi-Horng Liao

**Affiliations:** ^1^Bachelor Program in Digital Media and Technology, Tzu Chi University, Hualien City, Taiwan; ^2^Media Production and Education Center, Tzu Chi University, Hualien City, Taiwan

**Keywords:** pro-environmental behavior (PEB), social impact theory, theory of planned behavior (TPB), artificial neural networks (ANN), variance based structural equation modeling (VBSEM)

## Abstract

**Introduction:**

This study investigates the impact of social media on pro-environmental behavior (PEB) through the lenses of the Theory of Planned Behavior (TPB) and Social Impact Theory. The research aims to elucidate how social media influences Environmental Attitude (EA) and Subjective Norms (SN), and how these factors contribute to Behavioral Intentions (BI) that ultimately affect PEB. Additionally, it examines the moderating effect of Perceived Behavioral Control (PBC) on the relationship between BI and PEB.

**Methods:**

To explore these relationships, the study employs a dual methodological approach using Variance-Based Structural Equation Modeling (VBSEM) and Artificial Neural Networks (ANN). Data were collected from two distinct samples: 1200 participants from Taiwan for the SEM analysis and 602 respondents for the ANN study. SEM was utilized to explore causal relationships, while ANN was employed to enhance predictive accuracy.

**Results:**

The SEM analysis reveals that social media significantly affects both EA and SN, except for Social Networking Site Involvement (SNSI), which does not significantly impact EA. Additionally, the findings indicate that BI mediates the relationship between EA and PEB. However, BI does not mediate the SN-PEB relationship, and the link between SN and BI is found to be non-significant. Empirical evidence also suggests that PBC moderates the BI-PEB relationship, with a stronger influence observed under higher levels of PBC and a weaker influence under lower levels.

**Discussion:**

These results underscore the complex dynamics between social media factors and pro-environmental behavior. The study concludes that while social media plays a significant role in shaping EA and SN, its impact on EA is not mediated by SNSI. Furthermore, PBC significantly moderates the BI-PEB relationship, highlighting its critical role in PEB. The discussion addresses the implications of these findings, acknowledges the limitations encountered, and suggests potential avenues for future research.

## 1 Introduction

Climate change and pollution, urgent and interlinked environmental issues, pose a global concern. Pollution, a significant contributor to greenhouse gas emissions, accelerates climate change, leading to an alarming increase in the frequency and intensity of natural disasters worldwide. Lee A. R. et al. (2020) and Lee C. H. et al. (2020) documented a stark rise in environmental damage and losses, primarily attributed to climate change and pollution. For instance, Bowen and Ebi (2017) highlighted a dramatic surge in global natural disasters, including heatwaves, cold spells, and flash floods, all linked to severe climate shifts. The emission of greenhouse gases, a primary cause of climate change, is triggering widespread alterations in weather patterns influenced by both natural phenomena and human activities (Calista and Yenni, 2023). Consequently, weather patterns exhibit heightened severity and unpredictability. Taiwan, located in a high-risk zone for natural disasters such as earthquakes, typhoons, floods, and landslides, currently grapples with the challenges posed by climate change (Huang et al., 2016; Bowen and Ebi, 2017). To mitigate the exacerbation of climate change effects on Earth, it becomes imperative for individuals to undertake actions aimed at mitigating environmental concerns. Encouraging public involvement in environmentally beneficial actions is pivotal to fostering environmental awareness. This necessitates researchers and organizations to comprehend strategies for motivating and inspiring the public to engage in eco-friendly behaviors.

The Batson Model of Altruistic and Prosocial Behavior (Batson and Powell, 2003; Batson, 2014) demonstrates that behaviors and attitudes toward various subjects and objects can be enhanced by inducing empathy, an emotional response aligned with the perceived welfare of another. Empathy involves the ability to attribute mental states to another person and includes an appropriate affective response in the observer to the other person’s mental state (Baron-Cohen and Wheelwright, 2004; Ienna et al., 2022). Numerous studies have shown that induced empathy effectively improves attitudes toward people with AIDS, the homeless, and racial and ethnic minorities (Finlay and Stephan, 2000). Individuals with high levels of empathy exhibit stronger pro-environmental attitudes and behaviors compared to those with lower levels of empathy (Ienna et al., 2022). In this context, research suggests that inducing empathy may be a powerful technique for fostering more responsible environmental attitudes. Empathizing with nature can motivate conservation behavior and strengthen the belief in the effectiveness of one’s actions in aiding the natural environment. Thus, environmental attitudes and behaviors are significantly influenced by one’s empathy toward environmental conservation. Proponents of the primacy of empathy in conservation efforts, such as Sobel (1996), assert that conservation efforts must begin with empathy. This empathy can be cultivated through awareness generated by various communication sources.

Past research indicates that media platforms effectively disseminate information to the public, raising awareness about environmental issues and potential remedies. Scholars have recently shown a keen interest in exploring the relationship between media and pro-environmental behaviors (Xu and Han, 2019). Previous studies have established a relationship between mass media channels (e.g., radio, television, and newspapers) and pro-environmental behaviors. For instance, Huang (2016) demonstrated that the channels individuals use to gather information about global warming, primarily television, newspapers, and the Internet, significantly influence their environmentally friendly actions. Similarly, Trivedi et al. (2018) found that media negatively impacts internal environmental attitudes, consequently influencing green purchasing behavior. Mass media also shapes pro-environmental behavior by molding the public’s subjective identification of social norms.

Despite its prominence, social media has received limited attention in previous studies. However, numerous businesses utilize social media platforms like messaging sites, news websites, and short video platforms to promote eco-friendly consumption. Engagement and collaboration with others are promoted on social media platforms (Xie and Madni, 2023). Social media platforms use mobile and web technologies to create interactive environments for user-generated content, collaboration, and communication. Although individuals increasingly rely on social media platforms and spend significant time on them (Koay et al., 2020), scholarly attention on the relationship between media usage and pro-environmental behavior has predominantly focused on mass media (Xu and Han, 2019). Few studies have explored the role of social media in environmental conservation despite its immense potential to disseminate social causes digitally (Sujata et al., 2019). Despite the popularity of the growing digital media platform, as a social mobilization tool for direct action, existing research primarily views social media as a communication tool for influencing behavioral change, with a limited examination of its role in promoting environmental protection behavior (Sujata et al., 2019).

This study investigates how social media factors can promote pro-environmental behavior by examining attitudes toward environmental protection and subjective norms, employing a blend of social impact theory and the theory of planned behavior (TPB). The research contributes to the existing knowledge by comprehensively understanding how social media factors can encourage pro-environmental behavior. While prior studies primarily focus on the influence of mass media on pro-environmental behavior, this research delves into the specific influence of social media on environmental awareness and the underlying factors driving this influence. Furthermore, the study seeks to explore the role of attitudes in shaping the relationship between social media factors and pro-environmental behavior. By addressing the attitude-behavior gap, this research aims to provide valuable insights for environmental protection initiatives, guiding the development of effective social media campaigns to instigate positive changes in environmental behavior.

The current research aims to achieve the following objectives: (1) Explore the indirect relationships between social media factors and pro-environmental behavior, particularly through attitudes toward environmental information and subjective norms, and assess their impact on behavioral intention. (2) Investigate the mediating role of behavioral intention in the relationship between attitude and subjective norms on pro-environmental behavior. (3) Analyze the moderating effect of perceived behavioral control on the relationship between behavioral intention and pro-environmental behavior.

## 2 Literature review and hypotheses development

The following section delineates the constructs under examination, which encompasses social media exposure (SME), social media usage (SMU), user-generated content (UGC), social networking site involvement (SNSI), online interpersonal influence (OIP), Environmental Attitude (EA), subjective norms (SN), Behavioral Intention (BI), perceived behavioral control (PBC), and pro-environmental behavior (PEB). Additionally, this section furnishes a theoretical foundation to elucidate the rationale behind the relationships among these constructs.

### 2.1 Theoretical background

#### 2.1.1 Social impact theory

Social impact theory, proposed by Latané (1981), suggests that the effectiveness of social influence depends on three social factors: the number of people exerting influence, the immediacy or proximity of the source to the target, and the nature of the relationships between the source and the receiver. According to this theory, individual behaviors (the target) are shaped and modified by these social forces emanating from others (the sources). Essentially, as these factors increase, the impact on the target’s behavior becomes more pronounced. The theory explains how close sources form and influence opinions and how social influence is more likely to occur with factors such as number, immediacy, and strength (Latané, 1996). Various studies corroborate that larger social groups and more immediate sources exert a stronger social impact (Perez-Vega et al., 2016). For instance, research by Ding et al. (2017) indicates that a larger group of unfamiliar individuals can slightly enhance the likelihood of convincing someone to make a donation, compared to a smaller group, assuming both groups are equally significant and immediate. This implies that heightened social influence within a target population can foster increased pro-environmental behavior, as the pressure from more sources and closer proximity intensifies the impact on individuals’ actions. Bedard and Tolmie (2018) suggest using social impact theory to study how social media can raise environmental awareness and promote pro-environmental behavior. With extensive user populations and intimate virtual connections, social media is a fertile ground for studying social influence dynamics. Additionally, Chung and Austria (2010) note that social media goes beyond geographical and temporal barriers, facilitating information exchange and relationship-building without face-to-face interaction.

#### 2.1.2 Theory of planned behavior

Developed in 1985 by Ajzen, the Theory of Planned Behavior (TPB) explains human behavior within a social-psychological framework (Ajzen, 1985). It states that behavior is influenced by individuals’ conscious intentions to undertake specific actions based on their intentionality (Steg and Nordlund, 2018). The theory includes variables like attitude, subjective norm, perceived behavioral control, intention, and actual behavior (Groth et al., 2018). According to TPB, higher levels of positive attitude, subjective norm, and behavioral control increase the likelihood of behavior. The model suggests that all elements indirectly impact behavior through attitudes, norms, and behavior control (Steg and Nordlund, 2018).

#### 2.1.3 Pro-environmental behavior

Referring to purposeful actions aimed at mitigating the negative impact of human behavior on the environment (Kollmuss and Agyeman, 2002), the development of pro-environmental behavior is deemed crucial, as it signifies an individual’s inclination, awareness, and readiness to engage in environmental action (Suárez-Perales et al., 2021). Hence, motivating and inspiring the general public to adopt environmentally friendly practices is imperative, given that many environmental challenges stem from human activities (Liu et al., 2019). Research indicates that social media can influence pro-environmental behavior by indirectly altering individuals’ attitudes toward environmental information and subjective norms (Li and Noor, 2022).

### 2.2 Hypothesis development

#### 2.2.1 Social media usage, pro-environmental attitude, and subjective norms

Social media usage determines the amount of time individuals spend on social media, whether it be on a daily or weekly basis. It also addresses the level of user engagement and participation, including the quantity of social media group memberships, friends, and posts written and read (Zafar et al., 2021). Social media connects individuals with shared interests, facilitating information exchange, content creation, collaboration, and dissemination within online communities (Sujata et al., 2019). Users can communicate directly, form communities, and engage with like-minded individuals, facilitating minimal effort and cost (Carpenter et al., 2016). Social media users often perceive themselves as capable of affecting positive change (Groth et al., 2018).

Attitude is important in environmental contexts where individuals form attitudes toward the natural environment (Milfont and Duckitt, 2010). Positive attitudes often predict engagement, with individuals who have positive outlooks more likely to take action. Attitude toward a behavior is a person’s positive or negative opinion of the behavior (Ajzen, 1991). In environmental concerns, a pro-environmental attitude reflects care for the environment and support for its protection (Tian et al., 2020). It is a person’s concern for the natural environment and reflects prevailing attitudes and opinions toward the ecological environment. Social media usage can significantly influence environmental attitudes, fostering positive feedback loops that promote social benefits and well-being (Sujata et al., 2019) as individuals boost their self-esteem by sharing positive information about themselves with their friends and followers (Junsheng et al., 2019). It can also shape audience attitudes toward environmental considerations, such as eco-friendly packaging (Trivedi et al., 2018). Social media use can influence environmental attitudes, lifestyles, and consumption patterns (Bedard and Tolmie, 2018). Social impact theory suggests that social media platforms facilitate virtual connections despite face-to-face interaction (Chung and Austria, 2010).

Regarding subjective norms, which encompass societal expectations influencing behavior, social media usage significantly affects individuals’ perceptions of social acceptability (Ostic et al., 2021). In another study by Xie and Madni (2023) which investigated the impact of social media usage on the green purchase intentions of young people in the presence of subjective norms and perceived green value, demonstrated that perceptions about green environment and subjective norms have a strong mediating impact on increasing the intentions of consumers for purchasing of green products. Similarly, Santoso (2021) discovered that social media usage has a positive and significant effect on subjective norms. Therefore, in this regard, exposure to various social influences, comparisons, feedback, influence of popular figures, and trends on social media platforms can shape individuals’ perceptions of societal norms and anticipated behaviors (Santoso, 2021). This aligns with social impact theory, suggesting that individuals are more likely to be influenced by close sources, with the likelihood of responding to social influence increasing with immediacy and strength (Latané, 1996). Thus, based on the above discussions, the study proposes the following hypotheses:

*Hypothesis 1a:* Social media usage positively relates to pro-environmental attitude.

*Hypothesis 1b:* Social media usage positively relates to subjective norms.

#### 2.2.2 Online interpersonal influence, pro-environmental attitude, and subjective norms

Traditional face-to-face communication and social influence have been acknowledged for impacting individual behavior (Khare, 2014). Similarly, online interpersonal influence on social media platforms has gained prominence (Bedard and Tolmie, 2018), allowing individuals to reach a vast audience with minimal effort and employ adaptable strategies. Online influence among peers is often more effective than traditional advertising due to high trust levels between individuals (Khare, 2014). Interpersonal influence involves persuading and convincing others to achieve a desired outcome (Cheah and Phau, 2011), emphasizing the importance of one’s digital network, including family, friends, coworkers, and other connections (Bedard and Tolmie, 2018). These friendship networks shape behavior by spreading information, influencing opinions, and providing access to new ideas (Valente and Saba, 1998). Interpersonal influence holds considerable power in shaping attitudes and behaviors, with individuals susceptible to the influence of their social networks, as posited by Bearden et al. (1989). Their two-dimensional scale reflects susceptibility to normative and informational influences, illustrating the significant role of social influence in behavior formation. Previous research has consistently demonstrated a positive association between interpersonal influence and attitudes. For instance, Minhas and Furqan (2023) found empirical evidence suggesting that interpersonal influence directly impacts environmental attitudes. They argued that individuals rely on information gathered from their social networks—comprising friends, family, and peers—to form attitudes toward the environment. Thus, it can be asserted with confidence that interpersonal influence significantly contributes to the development of environmental attitudes. Increased interaction and discourse within one’s social circle are correlated with a greater likelihood of adopting positive environmental attitudes. Similarly, social impact theory’s three factors (number, immediacy, and strength) can aid in predicting the potential impact of online interpersonal influence, as digital networks enable diverse creators to share their experiences and opinions (Chung and Austria, 2010). Due to frequent internet and social media use, individuals often feel connected to members within their online networks (Bedard and Tolmie, 2018). Based on the above rationale, the study proposes the following hypotheses:

*Hypothesis 2a:* Online interpersonal influence positively relates to a pro-environmental attitude.

*Hypothesis 2b:* Online interpersonal influence positively relates to subjective norms.

#### 2.2.3 Social networking site involvement, pro-environmental attitude, and subjective norms

Social networking sites (SNS) are virtual spaces for users to engage and communicate (Ballew et al., 2015), allowing them to establish connections and engage in various online activities (Boyd and Ellison, 2007). High engagement indicates a strong interest in online social activities, such as information-seeking and gaming (Chai et al., 2019). SNSs also serve as platforms for information exchange and social connection (Namisango and Kang, 2019), allowing users to discuss environmental topics and share perspectives with a broader audience (Borg et al., 2021). These interactions foster collaboration and enhance understanding of collective environmental management (Scholz and Wang, 2006). SNS facilitates rapid information sharing and idea exchange, potentially influencing attitudes and choices (Bottaro and Faraci, 2022).

In the context of social impact theory, as individuals within close social circles engage in SNS activities advocating for environmental protection, the likelihood of a change in pro-environmental attitudes increases (Chang et al., 2018). Thus, strong ties among SNS users established through involvement on these platforms are likely to influence pro-environmental attitudes. Hence, the study proposes the following hypothesis:

*Hypothesis 3a:* SNS involvement positively relates to pro-environmental attitude.

Furthermore, SNSs provide a platform for users to observe societal expectations regarding values, norms, and beliefs, potentially influencing their perspectives on environmental issues (Han et al., 2018; Park, 2020). Engaging in environmental conversations on social media platforms can lead users to conform to subjective norms due to social pressure, particularly when environmental topics are discussed by their peers (Han et al., 2018). Additionally, SNSIs facilitate connections among individuals, including friends and family, fostering the creation of subjective norms through social persuasion. For example, frequent sharing of specific actions or thoughts by one’s friends can influence perceptions of what is expected in social situations. Consequently, the study proposes the following hypothesis:

*Hypothesis 3b:* SNS involvement positively relates to subjective norms.

#### 2.2.4 Exposure to social media content, pro-environmental attitude, and subjective norms

Media exposure refers to the degree to which individuals receive messages or media materials, whether or not they are sufficiently heard to be recognized (Slater, 2004). Social media exposure pertains to the visibility and accessibility of content or information on various platforms, encompassing how individuals, brands, or ideas are exposed to audiences through social media channels. The current study operationalizes exposure to social media content as the quantity of information heard or viewed on social media platforms regarding environmental protection. Informed individuals are better equipped to comprehend environmental issues, potentially motivating them to engage in pro-environmental activities (Shah et al., 2021). Traditional media campaigns on societal issues have been shown to influence people’s attitudes and behaviors when exposed to campaign messages (Liu et al., 2023).

The current study highlights the role of social media in shaping individual understanding of environmental issues and societal norms (Shah et al., 2021). Extensive media coverage increases environmental awareness, and the presentation of information on social media impacts audience responses and perceptions (Lee, 2011). Factors like the strength, immediacy, and abundance of information sources influence the impact of social media on environmental protection attitudes (Hu et al., 2023). In light of these considerations, the study proposes the following hypothesis:

*Hypothesis 4a:* Exposure to social media environmental content positively relates to a pro-environmental attitude.

Furthermore, social media exposure to environmental content and subjective norms can lead to individuals making subjective evaluations of green practices (Kang et al., 2013). Social comparisons can result in adopting certain behaviors based on others’ approval or disapproval (Jiang and Ngien, 2020). For example, if friends support environmentally friendly behaviors, individuals may need to adopt them to be accepted or viewed positively by others. Furthermore, social media exposure allows for the rapid spread of information and ideas among peer groups, leading to the perception of socially acceptable behaviors (Khalaf et al., 2023). This perception can influence subjective norms, as individuals may feel pressured to conform to the behaviors they see in their social circles online. Social media platforms often display content that mirrors popular opinions and behaviors (Yang et al., 2023), making individuals feel compelled to conform to these standards. Therefore, based on the above evidence, the following hypothesis is proposed:

*Hypothesis 4b:* Exposure to social media environmental content positively relates to subjective norms.

#### 2.2.5 Social media user-generated content, pro-environmental attitude, and subjective norms

User-generated content on social media platforms is gaining popularity as it unites online communities by allowing individuals to share thoughts, experiences, and perspectives (Wyles et al., 2019). This content, including images and tags, reflects personal values and can serve as valuable information for long-term behavior change (Chung and Koo, 2015). Social media allows users to co-create, share, discuss, and modify content, leading to increased engagement and sustainable behavioral change (Shaw, 2016). Studies in tourism show that UGC positively impacts destination image, attitudes, and intentions to visit (Kim et al., 2017). Similarly, Sultan et al. (2020), in their study aimed at understanding the effect of UGC on the responsible environmental behavior of travelers through environmental concerns and attitudes, demonstrated that cognitive and affective triggers of user-generated content influence travelers’ environmental concerns and attitudes, making a significant contribution to shaping responsible environmental behavior. In this regard, it can be safely stated that UGC can lead to the making up of positive attitudes among individuals. Therefore, concerning the above argument, the study proposes the following hypothesis:

*Hypothesis 5a:* Social media UGC positively relates to pro-environmental attitude.

Social media user-generated content (UGC) can also influence subjective norms by validating social behavior and influencing what is considered appropriate or attractive in a social circle. Social validation features like likes, comments, and shares can reinforce the belief that actions or thoughts are socially accepted, leading to others following these norms (Ballara, 2023). In line with social impact theory, the strength of influence, immediacy, and size of the influencing group determine an individual’s tendency to conform to social pressure, influencing subjective norms. Overall, social media UGC plays a crucial role in shaping social norms (Latané, 1981). In this case, social media often displays user-generated content from friends, family, celebrities, or influencers, with varying levels of importance to each person. The more a user perceives the content source to be strong, the more likely they are to follow the subjective norms expressed in that content. Social media provides instant access to user-generated content, impacting subjective norms. Additionally, multiple sources are exposed simultaneously (Luca, 2015). As more people share an opinion or behavior, it becomes the standard and influences others. This leads to social proof, where people think others’ behavior is the proper response, reinforcing subjective norms. Therefore, based on the above arguments, the following hypothesis has been proposed:

*Hypothesis 5b:* Social media UGC positively relates to subjective norms.

#### 2.2.6 Attitude toward pro-environmental behavior, subjective norms, and environmental behavioral intentions

Moisander (2007) defined behavioral intentions as strong internal stimuli that are often understood as the cause of behaviors. According to the TPB, intentions indirectly predict different behaviors (Ajzen, 1991; Liu et al., 2020). Environmental behavioral intention is the perceived subjective of a person participating in environmental behaviors, reflecting the disposition of the person to participate in a specific environmental behavior (Kaiser and Gutscher, 2003). According to the TPB, people are more likely to have intentions to engage in behaviors that they perceive positively due to their attitudes (Bosnjak et al., 2020). Research studies have documented how positive attitudes contribute to intentions to engage in altruistic, pro-environmental, and pro-social behaviors (Smith and McSweeney, 2007). For example, positive attitudes toward a social issue can forecast charitable donations and volunteering related to that issue (Greenslade and White, 2005). Similarly, positive attitudes toward pro-environmental actions may indicate favorable problem-solving behaviors. The more positive the attitude and social norms, the stronger the behavioral control, and the more likely individuals are to perform the behavior (Ajzen, 1991). Research by Yadav and Pathak (2016) and Liu et al. (2020) supports the efficacy of the TPB in predicting intentions to purchase eco-friendly products, emphasizing the impact of attitudes. Hence, in reference to the arguments mentioned above, the study hypothesizes that:

*Hypothesis 6a:* A pro-environmental attitude positively and significantly affects behavioral intention.

The TPB suggests that individuals’ intentions are influenced by their beliefs and expectations of others (Kan and Fabrigar, 2017). People are more likely to plan a behavior if they believe their important people approve of it, while a lack of encouragement may decrease their intention. Based on these expectations, subjective norms are more likely to influence behavioral intentions. Research has shown a relationship between subjective norms and intentions, such as the positive impact of using bikes sustainably (Si et al., 2020). Similarly, Yadav and Pathak (2016) found that the subjective norm of young consumers influences their intentions to purchase environmentally friendly products. Furthermore, Dalila et al. (2020) and Shanmugavel and Balakrishnan (2023) demonstrated that subjective norms positively influence the behavioral intention toward e-vehicles. In this light, research has shown that individuals who believe that important people in their lives strongly support environmentally friendly behavior are more likely to have a strong intention to engage in such behavior (Ateş, 2020). Therefore, based on the above narration and empirical evidence, the following hypothesis has been proposed:

*Hypothesis 6b:* Subjective norms are positively and significantly related to behavioral intention.

#### 2.2.7 Pro-environmental attitude, subjective norms, and pro-environmental behavior

The TPB suggests positive attitudes can predict behavioral intentions to engage in certain behaviors (Ajzen, 1991). People with a positive pro-environmental attitude are more likely to protect the environment and participate in various pro-environmental behaviors (Bamberg and Möser, 2007; Tian et al., 2020). This aligns with previous research, which suggests that a positive attitude directly influences employees’ actions, such as taking initiative in pro-environmental efforts and completing environmentally friendly tasks (Bissing‐Olson et al., 2013). Additionally, clients with more favorable environmental attitudes prefer environmentally friendly products (Malik and Singhal, 2017). Hence, the study proposes the following hypothesis:

*Hypothesis 7a:* Pro-environmental attitude positively relates to pro-environmental behavior.

On the other hand, social psychology research indicates that norms and values can influence behavior, with family and peers playing a significant role in shaping beliefs, attitudes, and behaviors (Schultz et al., 2008; Lee, 2011). Young et al. (2017) found that individuals’ intentions to participate in specific behaviors are often influenced by their perceptions of their peers’ involvement or endorsement. Additionally, behaviors they believe are appropriate within their social group, such as environmentally friendly activities, can influence individuals to conform to these norms (McDonald and Crandall, 2015). Therefore, this leads the study to propose the following hypothesis:

*Hypothesis 7b:* Subjective norms positively relate to pro-environmental behavior.

#### 2.2.8 Behavioral intention and pro-environmental behavior

Ajzen (1991) posited that intention is a crucial antecedent to behavior, and individuals tend to perform actual behaviors when their intentions become stronger (Ajzen and Fishbein, 2000). Studies have shown positive relationships between intentions and actual behaviors, particularly in eco-friendly purchases, altruistic behavior, and pro-environmental purchasing behavior (Onel, 2017). In his study, Intention was confirmed to be a good predictor of actual behavior, making it a crucial determinant in explaining consumers’ actual eco-friendly purchase behaviors. In this regard, we expect behavioral intentions to influence pro-environmental behavior; hence, we propose the following hypothesis:

*Hypothesis 8:* Behavioral intentions positively relate to pro-environmental behavior.

#### 2.2.9 The mediating effect of behavioral intention in the relationships among environmental attitudes, subjective norms, and pro-environmental behavior

Ajzen (1991), through the Theory of Planned Behavior (TPB), suggested that intentions play a crucial intermediary role in predicting behaviors across various factors. The research highlights how individuals’ attitudes, subjective norms, and perceived behavioral control concerning environmental issues shape their intentions, which in turn, influence their pro-environmental behaviors. Thus, intentions are expected to mediate the relationships between environmental attitudes, subjective norms, and actual pro-environmental actions. Based on this theoretical framework, the study proposes the following hypotheses:

*Hypothesis 9a:* Behavioral intentions mediate the direct relationship between environmental attitude and pro-environmental behavior.

*Hypothesis 9b:* Behavioral intentions mediate the direct relationship between subjective norms and pro-environmental behavior.

#### 2.2.10 The moderating role of perceived behavioral control in the relationship between behavioral intention and pro-environmental behavior

PBC is a fundamental concept in the TPB framework. It refers to an individual’s perception of how easy or difficult it is to perform a given behavior (Ajzen, 1991). PBC reflects the confidence an individual has in their capability to execute the behavior, which significantly impacts their ability to convert intentions into actions. Individuals with higher perceived behavioral control feel more empowered and are thus more likely to follow through on their intentions (Luszczynska et al., 2011).

In the context of TPB, PBC not only influences intentions but can also serve as a moderator in the relationship between BI and actual behavior. Research indicates that individuals who perceive greater control over their actions are more likely to translate their intentions into behaviors, effectively bridging the gap between what they intend to do and what they actually do (Oh et al., 2020).

For instance, when people believe they have the resources, opportunities, and skills to perform a behavior, the impact of persuasive messages on their intentions to act is amplified. This relationship underscores the significant role that PBC plays in shaping both the formation of intentions and the execution of behaviors. Given the substantial influence of PBC on attitudes, subjective norms, intentions, and behaviors, this study posits the following hypothesis:

*Hypothesis 10:* Perceived Behavioral Control moderates the relationship between Behavioral Intentions and Pro-Environmental Behavior, which is stronger when there is greater perceived behavioral control.

**Figure 1:**
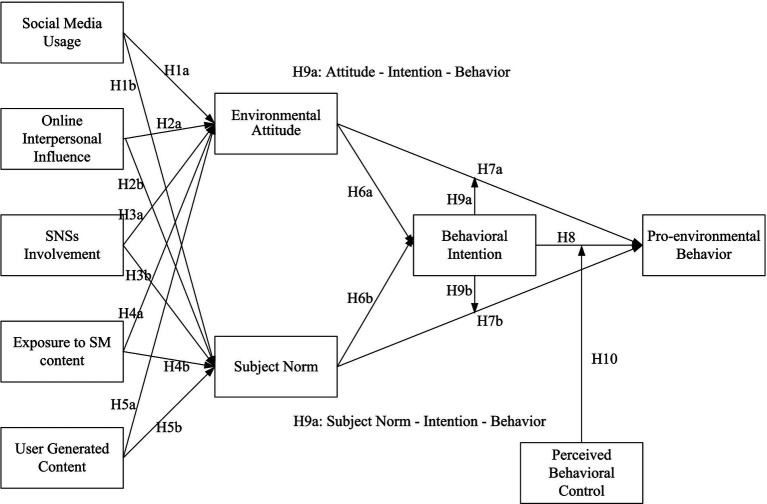
Research framework.

This hypothesis suggests that the strength of the link between an individual’s intentions to engage in pro-environmental behaviors and their actual engagement in these behaviors is significantly influenced by their perceived level of control over these behaviors. To illustrate the hypothesized relationships within the SEM framework, Figure 1 presents the research model. It highlights how PBC is expected to enhance the effect of BI on PEB.

## 3 Methodology

The study employs a unique approach by integrating SEM with ANN. The participants are detailed, along with the cross-sectional method used, sample size determination, questionnaire creation, measurement items, and data analysis methodology. The study also discusses the ANN model.

### 3.1 Structural equation modeling

SEM uses multiple variables to forecast results by analyzing regression and integrating statistical techniques like factor analysis and path analysis. It validates research findings by analyzing data. Lin et al. (2020) uses it to analyze variables’ relationships and confirm theories. According to Fan et al. (2016), suggested actions for creating SEM include creating a framework, quantifying factors with visuals, evaluating the model, and making improvements.

#### 3.1.1 Research participants

The study collected data from Taiwanese adults aged 18 and older who regularly use social media platforms. Online surveys were distributed via Google Forms, and participants were invited via Facebook Messenger, WhatsApp, and Line. Convenient sampling, a nonprobability method, was used to select participants based on accessibility and willingness (Taherdoost, 2016). However, it has drawbacks like selection bias and lack of representativeness (Stratton, 2021). The survey focused on social media factors and gathering demographic information such as gender, age, education, and employment status in two sections.

The dataset used for analysis consisted of responses provided by 1,200 participants. As presented in Table 1, participants were classified based on their gender as either male (1) or female (2). In the final sample, there were 386 male participants, accounting for 32.2% of the total, and 814 female participants, making up 67.8%. Participants were asked to indicate their age bracket, with the age group of 18–25 being the most common, chosen by 762 individuals (63.5%). In contrast, the age group of 26–40 had the smallest number of participants, with only 99 individuals (8.3%). In relation to education, participants were categorized according to their most advanced level of education. Individuals with bachelor’s degrees made up the largest group, comprising 68.0% of the total participants, with a frequency of 816. On the other hand, the smallest group, with a frequency of 384 participants, consisted of those with post-graduate degrees. Most participants, 762 (63.5%), were students, while the smallest group consisted of 162 (13.5%) who were unemployed.

**Table 1:** Sample Structure.

Demographics	Frequency	Percentage (%)
**Gender**		
Male (1)	386	32.2
Female (2)	814	67.8
**Age**		
18–25 (1)	762	63.5
26–40 (2)	99	8.3
41–55 (3)	223	18.6
56 or more (4)	116	9.7
**Education**		
Elementary (1)	0	0
High School (2)	0	0
Bachelor (3)	816	68.0
Postgraduate (4)	384	32.0
**Employment status**		
1-Employed	162	13.5
2-Self-employed	276	23.0
3-Not employed	0	0
4-Student	762	63.5

#### 3.1.2 Measurement items

The questionnaire design was primarily based on multiple-item measurement scales adapted from previous research on SME, SMU, OIP, SNSI, UGC, EA, SN, BI, PBC, and PEB. All measurement items were developed using 5-point rating scales with the following anchors: 1 = strongly disagree, 2 = Disagree, 3 = Neutral, 4 = Agree, and 5 = Strongly agree, except for the SME and SNI constructs, which had the following anchors: 1 = Never, 2 = Rarely, 3 = Sometimes, 4 = Often, 5 = Always and PBC with the following anchors; 1. Very difficult, 2 = Difficult, 3 = Neutral, 4 = Easy, 5 = Very easy. The research design of this study includes ten different variables, each containing a specific number of items. Certain expressions were modified to align more closely with the focus of this research.

SME was measured by asking participants to indicate how often they see, read, and hear environmental information on the following social media platforms: Facebook, Instagram, Twitter, YouTube, and WhatsApp (Zeballos Rivas et al., 2021). SMU was assessed by indicating their agreement with using social media platforms (Biswas, 2016). Some items include “I find it easy to use social media sites” and “Use of social media has enhanced my efficiency in social networking.” SNSI was measured on a 5-point Likert scale, with respondents indicating how often they are active on social networking sites and involved in various activities, for example, “Reading/seeing posts on environmental sustainability” and “Liking posts on environmental sustainability” (Groth et al., 2018). OIP was judged by a 9-measurement scale adapted from Bearden et al. (1989) and Fan et al. (2019). UGC was assessed by 6-items adapted from Kim and Johnson (2016). Sample items include “The postings that appear on my social media platforms describe environmental protection actions” and “The postings that appear on my social media platforms describe benefits of environmental protection. Participants assessed EA using a 7-measurement scale item adapted from Lee (2011) with sample items “It is essential to promote pro-environmental behavior” and “I strongly support that more environmental protection works are needed.” Four items were used to measure SN (Al-Swidi et al., 2014). Two measurement items from Ateş (2020) were used for BI to assess the variable. Likewise, participants responded to two measurement items adapted from Ateş (2020) to measure PBC. Lastly, PEB was assessed by eight measurement items adapted from Mónus (2021) and Markowitz et al. (2012).

#### 3.1.3 Data analytical tools

VBSEM is the primary method used to analyze construct relationships in this study. Jannoo et al. (2014) used PLS-SEM and SPSS software for accurate estimates with sample sizes above 50. PLS-SEM is ideal for complex structural models with multiple constructs, indicators, and relationships. This study has ten constructs with 59 items and multiple model relationships. Hence, smart-PLS was selected. The data used in this study had distribution problems, so PLS-SEM was chosen as it is suitable for non-normal data and does not require specific distributional assumptions. Lastly, PLS-SEM provides solutions when CB-SEM produces unsatisfactory results (Sarstedt et al., 2016). PLS-SEM has greater chances of identifying significant connections when present in the population, giving it more statistical power than other methods (Sarstedt et al., 2019). In this study, PLS is robust against non-normal data distribution (Hew and Syed, 2017), as all variables in the model have *p*-values of the Kolmogorov–Smirnov statistics less than 0.05 (Tan et al., 2014). IBM SPSS 22 and the macro-PROCESS V4.2 analyzed mediation and moderation effects through bootstrapping.

### 3.2 Artificial neural network

Researchers can also use Artificial Neural Network (ANN) as an alternative method to CB-SEM and VB-SEM (Leong et al., 2019). ANN is a non-compensatory analytical technique with deep learning algorithms based on input, output, and hidden layers (Yang et al., 2022). It is considered the most versatile analytical method as it does not require multivariate assumptions or hypotheses (Hew et al., 2017). The minimum sample size for ANN can be calculated by multiplying input variables by 50, providing 50 times more adjustable parameters (Alwosheel et al., 2018). The study suggests combining SEM and ANN methodologies could offer a superior research approach. ANN uses artificial neurons in hidden layers to enhance prediction accuracy by connecting input and output data. SEM can examine causal relationships, while ANN can uncover linear and nonlinear relationships and learn through artificial intelligence mechanisms (Leong et al., 2019). SEM can examine intricate relationships between independent and dependent variables, including factors or measured variables. SEM is a confirmatory technique used to assess, test hypotheses, adapt existing models, or examine interconnected models (Sternad Zabukovšek et al., 2019). Both methods complement each other rather than conflict (Tabachnick and Fidell, 1983).

Thus, the study suggests that combining SEM and ANN for hypothesis testing and identifying nonlinear relationships in a non-compensatory model, using the RMSE value for predictive accuracy, is suitable (Hayat et al., 2021).

## 4 Results

### 4.1 SEM results

#### 4.1.1 Multicollinearity assessment

The study used Tolerance and Variance Inflation Factor (VIF) to check for multicollinearity issues. The results showed that all study constructs had VIF values less than 10 (Hair et al., 2019), indicating no multi-collinearity issues, with the highest VIF value being 2.409.

#### 4.1.2 Common method bias

The study used Harman’s single factor test to detect common method bias (CMB). The first factor explained 16.720% of the variance, indicating no serious problem with CMB, as the variance for the first factor was below 50% in the dataset (Fuller et al., 2016).

#### 4.1.3 Measurement model assessment

##### 4.1.3.1 Composite reliability, discriminant, and convergent validity

To obtain satisfactory reliability, the reliability coefficient or Cronbach’s alpha should reach a minimum of 0.7 or higher (Hair et al., 2010). The results obtained for the ten variables of the present study fulfill the required conditions of Cronbach’s alpha minimum value of 0.7: BI = 0.74, EA = 0.71, OIP = 0.71, PBC = 0.71, PEB = 0.71, SME = 0.80, SMU = 0.71, SN = 0.79, SNI = 0.75, and UGC = 0.75 indicating acceptable reliability.

Furthermore, Hair et al. (2014) stated that a composite reliability (CR) value greater than 0.7 guarantees the reliability of all constructs. Table 2 shows that the CRs range from 0.807 to 0.886, indicating a remarkably high-quality standard. Average variance extracted (AVE) quantifies how much of the variance in a construct can be attributed to the construct itself instead of measurement errors. According to Hair et al. (2010), values over 0.5 are considered acceptable when assessing the model’s constructs for convergent validity. Alternatively, Alarcón et al. (2015) stated that AVE values surpassing 0.4 may be deemed sufficient in different areas of study. This table also displays test results indicating that most AVE values surpass the 0.5 threshold.

**Table 2:** Means, standard deviations, and correlations.

Variables	Mean	SD	CR	AVE	1	2	3	4	5	6	7	8	9	10
1. SME	3.7	0.75	0.86	0.56	0.75									
2. SMU	3.6	0.51	0.82	0.42	−0.46^**^	0.65								
3. SNI	3.7	0.72	0.84	0.57	0.16^**^	−0.02	0.76							
4. OIP	3.6	0.52	0.81	0.41	0.12^**^	0.26^**^	0.09^**^	0.64						
5. UGC	3.8	0.56	0.84	0.50	−0.03	0.40^**^	0.03	0.69^**^	0.71					
6. EA	3.7	0.54	0.81	0.52	0.050	0.10^**^	0.03	0.58^**^	0.51^**^	0.72				
7. SN	3.6	0.78	0.86	0.61	−0.15^**^	0.43^**^	0.24^**^	0.32^**^	0.40^**^	0.18^**^	0.78			
8. BI	3.9	0.92	0.89	0.80	0.31^**^	−0.15^**^	0.40^**^	0.21^**^	0.10^**^	0.07^*^	−0.02	0.9		
9. PEB	4.1	0.53	0.82	0.46	0.11^**^	0.32^**^	0.03	0.62^**^	0.64^**^	0.46^**^	0.29^**^	0.20^**^	0.68	
10. PBC	4.3	0.76	0.87	0.77	0.62^**^	−0.20^**^	0.24^**^	0.28^**^	0.16^**^	0.14^**^	0.01	0.39^**^	0.27^**^	0.88

N = 1,200; ^*^ *p* < 0.05; ^**^ *p* < 0.01; ^***^ *p* < 0.001.Reliability coefficients are reported along the diagonal parentheses.

On the other hand, the AVEs for OIP, PEB, and SMU experienced a minor decline, falling just below 0.5 and settling at 0.41, 0.46, and 0.42, respectively. AVEs, which fall below the recommended level of 0.5, may be a conservative estimation of a measurement model’s validity. Fornell and Larcker (1981) argue that composite reliability alone can confirm construct convergent validity, even if over 50% of the variance is due to error. The composite reliability of all constructs is higher than the recommended level. Furthermore, the study evaluated discriminant validity between constructs using Fornell and Larcker (1981) suggestion. The correlation matrix showed higher values along the main diagonal (square root of AVE) than off the diagonal (bivariate correlations), indicating good discriminant validity of the model’s scales.

In summary, the reliability and validity results, as tested in the Smart-PLS 3, indicate that the constructs measurement items were reliable and valid. However, items with lower factor loadings were removed from the study, as they were considered less significant than the minimum requirement. For instance, items SMU1, SMU3, SMU7, SMU9, SNI1, OIP3, OIP8, OIP9, UGC1, EA6, EA7, and PEB1 and PEB3 were removed for social media usage, involvement, interpersonal influence, user-generated content, environmental attitude, and pro-environmental behavior.

##### 4.1.3.2 Confirmatory factor analysis

The study used Confirmatory Factor Analysis (CFA) in AMOS 21.0 to evaluate a ten-factor model’s validity and goodness of fit. The model showed satisfactory fitness, with a χ2 value of 1940.381 and degrees of freedom at 944. The CFI (0.937), IFI (0.937), TLI (0.931), and RMSEA (0.030) indicated a reasonable and acceptable fitness level (Jöreskog and Sörbom, 1993).

#### 4.1.4 Hypotheses testing

The study employed structural equation modeling to examine the research hypotheses describing relationships among constructs using Smart-PLS 3.5. The test results indicate the anticipated magnitude and relevance of the supposed linkages between groups of variables that either confirm or reject hypotheses. The path coefficients are evaluated on a scale ranging from negative to positive. Values closer to +1 indicate a stronger positive correlation, while values closer to −1 indicate a stronger negative correlation. The results in Table 3 indicate that all the relationships except (SN – > BI and SNI – > EA) were supported as their *p*-values were below 0.05.

**Table 3:** Results of examination of direct research hypotheses.

Variables	Path coefficients	*p* values	Remarks
BI→PEB	0.112	***	Supported
EA → BI	0.079	0.007	Supported
EA → PEB	0.389	***	Supported
OIP → EA	0.457	***	Supported
OIP → SN	0.066	0.043	Supported
SME → EA	0.057	0.023	Supported
SME → SN	0.055	0.043	Supported
SMU → EA	0.144	***	Supported
SMU → SN	0.304	***	Supported
SN → BI	−0.038	0.177	Not Supported
SN → PEB	0.222	***	Supported
SNI → EA	−0.007	0.739	Not Supported
SNI → SN	0.250	***	Supported
UGC → EA	0.260	***	Supported
UGC → SN	0.228	***	Supported

Model fitness: χ2 = 1940.381, df = 944, χ2/df = 2.0555, RMSEA = 0.03, CFI = 0.937, IFI = 0.937, TLI = 0.931.*N* = 1,200; ^*^ *p* ≤ 0.05; ^**^ *p* ≤ 0.01; ^***^ *p* ≤ 0.001.SME, social media exposure; SMU, social media usage; UGC, user-generated content; SNSI, social networking site involvement; OIP, online interpersonal influence; EA, environmental attitude; SN, subjective norms; BI, behavioral Intention; PEB, pro-environmental behavior.

**Figure 2:**
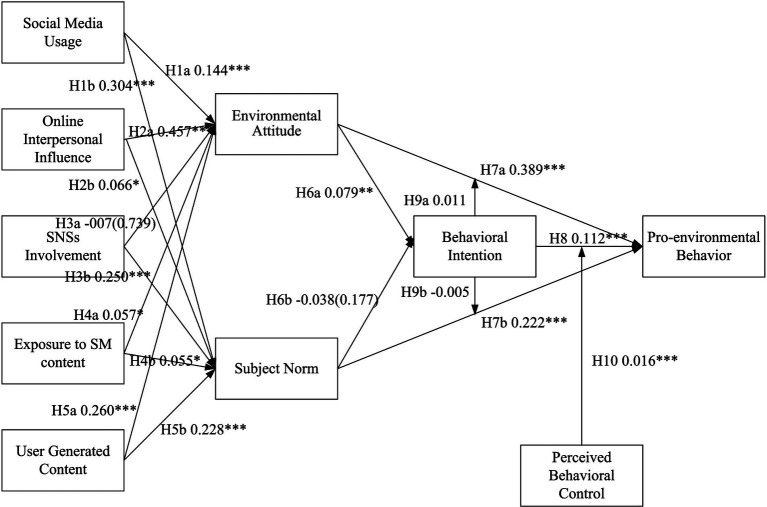
Path estimates for SEM.

Figure 2 illustrates the path estimates for the Structural Equation Modeling (SEM) analysis. This figure visually represents the relationships and strength of associations between variables, as the hypothesized model specifies. The path estimates provide critical insights into how the independent variables influence the dependent variables and help to understand the overall structural relationships within the model. Numbers in the parentheses indicate the *p*-values of the hypotheses while values outside the parentheses are the path estimates.

##### 4.1.4.1 Mediation analysis

To explore mediation effects, a bootstrap analysis was performed using SPSS with the PROCESS macro 4.1 (Model 4) (Hayes, 2012). Cheung and Lau (2008) suggest that for mediation effects to be significant, the 95% confidence interval (CI) for the estimates must exclude zero. As shown in Table 3, the analysis revealed one significant mediation pathway. The path from Environmental Attitude (EA) to Pro-Environmental Behavior (PEB) via Behavioral Intention (BI) had a 95% confidence interval that did not include zero (estimate = 0.011, 95% CI [0.002, 0.022]). This result supports Hypothesis 9a, confirming that Behavioral Intention mediates the relationship between Environmental Attitude and Pro-Environmental Behavior.

Conversely, the path from Subjective Norms (SN) to Pro-Environmental Behavior (PEB) through Behavioral Intention (BI) included zero within its 95% confidence interval (estimate = −0.005, 95% CI [−0.016, 0.007]). This finding indicates no significant mediation effect for this pathway, thus providing no support for Hypothesis 9b, which proposed that Behavioral Intention would mediate the relationship between Subjective Norms and Pro-Environmental Behavior.

##### 4.1.4.2 Moderation analysis

In examining Hypothesis 10, which posited that PBC moderates the relationship between BI and PEB, the analysis revealed a significant interaction effect. The moderation analysis identified a substantial interaction between PBC and BI on PEB (b = 0.016, *p* < 0.001), indicating that individuals with higher PBC exhibit a stronger influence of BI on PEB compared to those with lower PBC. The findings suggest that the strength of the relationship between BI and PEB is contingent upon the level of PBC. Specifically, as PBC increases, the impact of BI on PEB becomes more pronounced. Conversely, at lower levels of PBC, this relationship diminishes. This evidence supports Hypothesis 10, affirming that PBC significantly moderates the relationship between BI and PEB.

**Figure 3:**
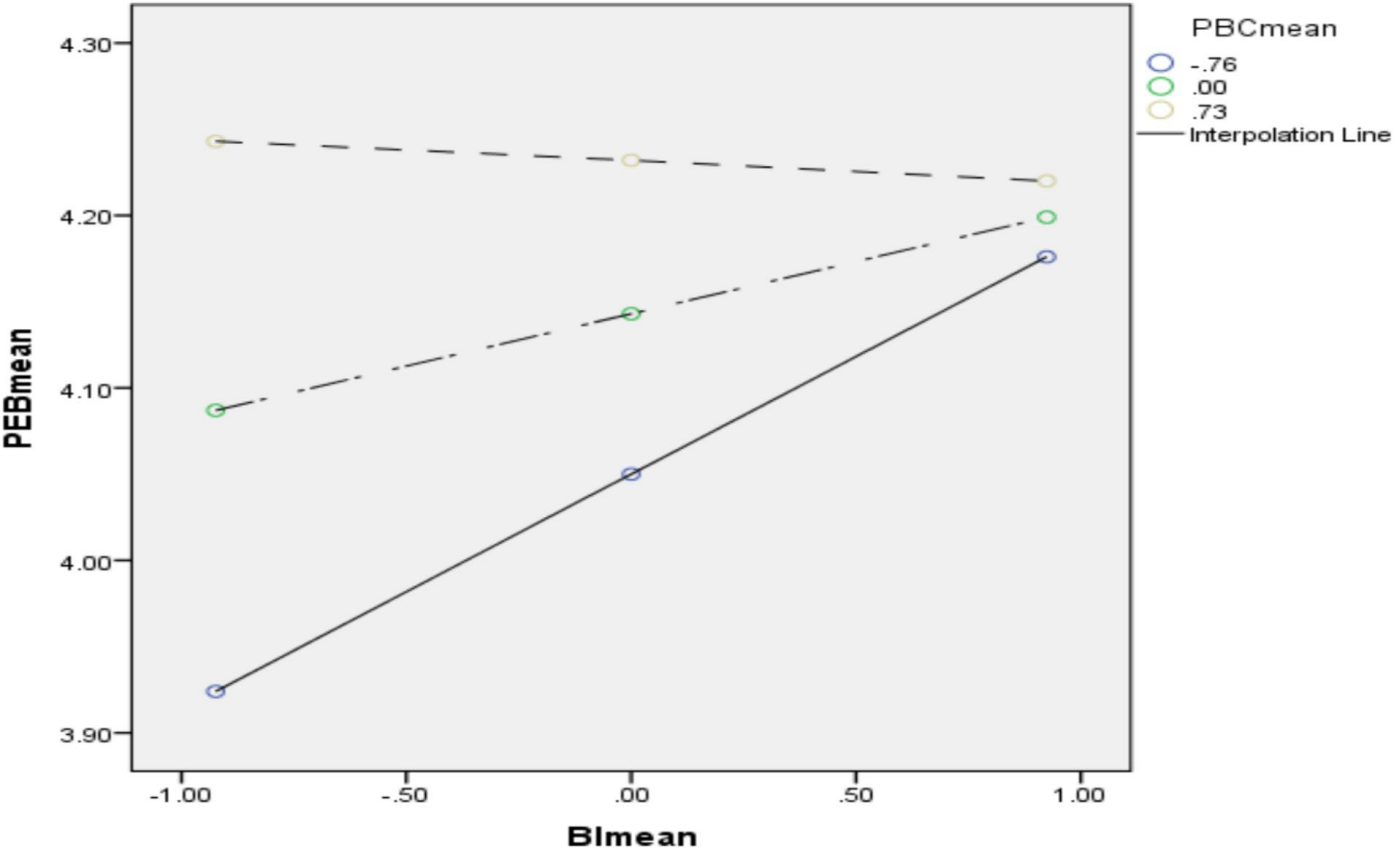
Moderation plot. SME, social media exposure; SMU, social media usage; OIP, online interpersonal influence; UGC, user-generated content; EA, environmental attitude.

Figure 3 provides a moderation plot that illustrates the varying strength of the BI and PEB relationship across different PBC levels. The plot demonstrates that higher PBC enhances the effect of BI on engaging in pro-environmental behaviors, thereby corroborating the hypothesized interaction. The consistency between the interaction analysis and the moderation plot underscores the hypothesis that the relationship between BI and PEB is strengthened under higher levels of PBC. This is in contrast to the weaker relationship observed under lower levels of PBC. These results validate the proposed theoretical framework and highlight the critical role of PBC in influencing the translation of behavioral intentions into pro-environmental actions.

### 4.2 Artificial neural network results

This study employs SPSS 22 to simulate three ANN models for analyzing and interpreting data. These models are designed to evaluate various aspects of pro-environmental behavior and its determinants: Model A assesses EA using four specific factors. Model B evaluates EA with five variables. Model C measures PEB by considering EA, SN, and BI.

**Figure 4:**
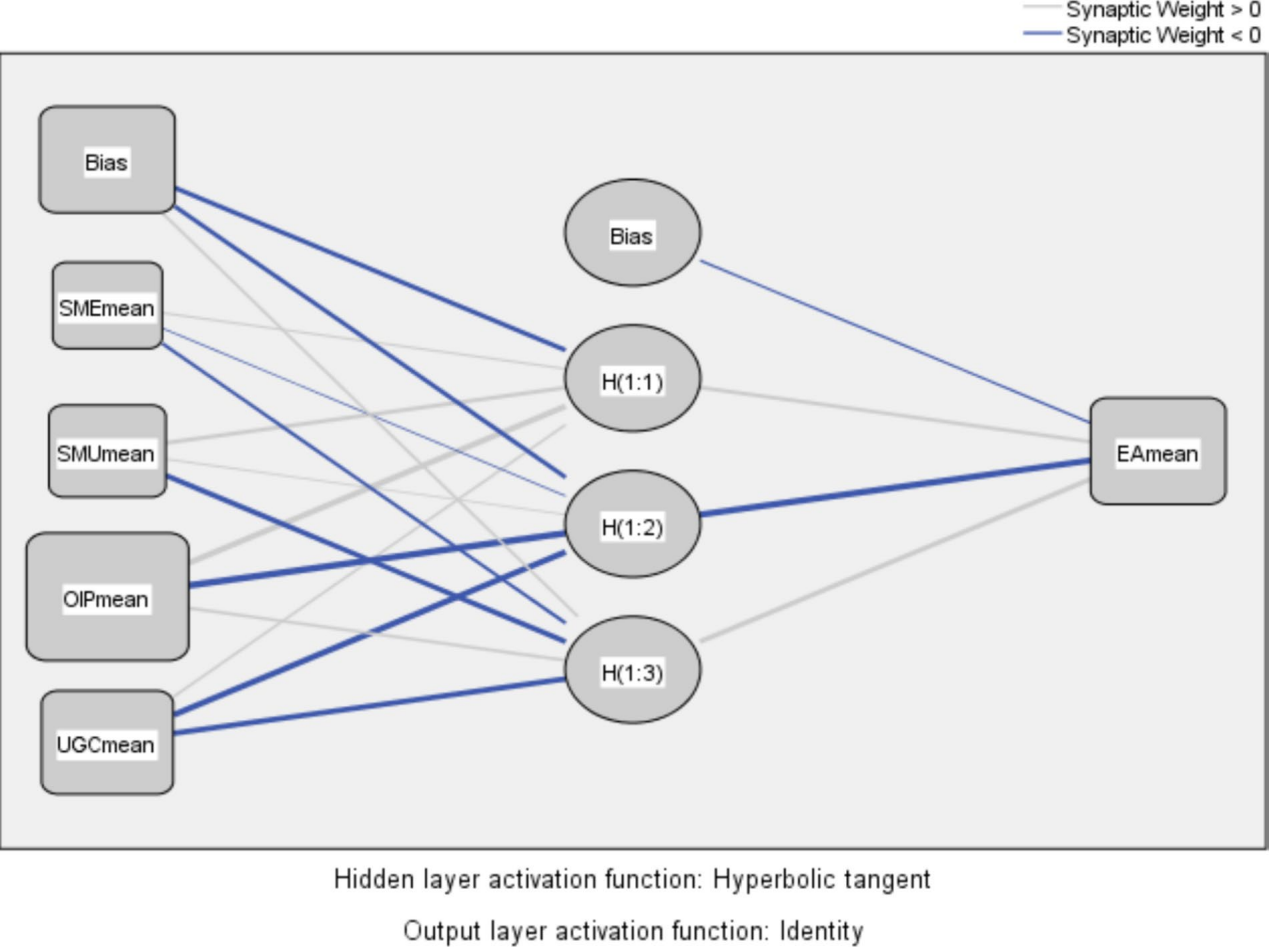
ANN model for EA output (model A).

The accuracy of these ANN models is determined using the Root Mean Square Error (RMSE) metric, following the methodology outlined by Yadav and Pathak (2016). Each ANN model incorporates a single hidden layer, with the number of neurons in this layer automatically generated by SPSS, as Leong et al. (2015) recommended. Figure 4 presents an ANN model for EA output (model A) representing as an example for other models.

Table 4 presents the average RMSE values for all three ANN models. These values range from 0.298 to 0.678 for the training sets and from 0.278 to 0.696 for the testing sets, using 70% of the data for network training and the remaining 30% for testing. The results indicate moderate accuracy and reliability in modeling the relationships between the independent and dependent variables, as Ooi and Tan (2016) suggested.

**Table 4:** RMSE values of artificial neural networks (*N* = 602).

	MODEL A		MODEL B		MODEL C	
Network	RMSE (Training)	RMSE (Testing)	RMSE (Training)	RMSE (Testing)	RMSE (Training)	RMSE (Testing)
ANN1	0.317	0.278	0.445	0.420	0.678	0.546
ANN2	0.298	0.332	0.462	0.494	0.660	0.673
ANN3	0.301	0.321	0.404	0.509	0.603	0.668
ANN4	0.304	0.311	0.403	0.606	0.590	0.591
ANN5	0.298	0.333	0.491	0.424	0.599	0.584
ANN6	0.313	0.296	0.468	0.417	0.610	0.638
ANN7	0.304	0.328	0.467	0.431	0.614	0.696
ANN8	0.323	0.279	0.462	0.490	0.637	0.611
ANN9	0.301	0.321	0.465	0.423	0.579	0.660
ANN10	0.335	0.282	0.430	0.453	0.583	0.576

Additionally, the outcomes derived from the Partial Least Squares Structural Equation Modeling (PLS-SEM) were compared to those from the ANN models to verify consistency and accuracy. As illustrated in Table 5, there is a discrepancy in the ranking of factors between Model A and Model B. However, Model C demonstrates identical factor rankings, suggesting a closer alignment in evaluating PEB.

**Table 5:** Comparison between PLS-SEM and ANN analysis.

PLS-Path	Path coefficient	Normalized relative importance	Ranking based in PLS-SEM	Ranking based in ANN	Remark
Model A					
SMU → EA	0.144	7.6%	3	4	Mismatch
OIP → EA	0.457	100%	1	1	Match
SME → EA	0.057	9.9%	4	3	Mismatch
UGC → EA	0.260	64.1%	2	2	Match
Model B					
SMU → SN	0.304	100%	1	1	Match
SNI → SN	0.250	71.1%	2	3	Mismatch
OIP → SN	0.066	19.9%	4	4	Match
SME → SN	0.055	14%	5	5	Match
UGC → SN	0.228	82%	3	2	Mismatch
Model C					
EA → PEB	0.389	100%	1	1	Match
BI→PEB	0.112	42%	3	3	Match
SN → PEB	0.222	73%	2	2	Match

SME, social media exposure; SMU, social media usage; UGC, user-generated content; SNSI, social networking site involvement; OIP, online interpersonal influence; EA, environmental attitude; SN, subjective norms; BI, behavioral Intention; PEB, pro-environmental behavior.

In Model A, both ANN and SEM approaches identified interpersonal influence as the most significant determinant of EA, closely followed by user-generated content. SME and SMU demonstrated significantly weaker influences. Model B revealed that SMU is the most critical predictor of SN according to both ANN and SEM analyses. The less influential determinants of SN are identified as OIP and SME. In Model C, the results indicate that PEB is predominantly influenced by EA, followed by SN. BI exhibited a comparatively weaker impact in both the ANN and SEM methodologies.

## 5 Discussion

This study explores the influence of social media factors on pro-environmental behavior through attitudes and subjective norms, using the theories of planned behaviors and social impact. It examines attitudes toward environmental protection and subjective norms and their role in shaping behavioral intentions. The study also examines how BI affects the link among attitude, subjective norms, and PEB and how PBC moderates the relationship between BI and PEB. The findings have significant theoretical and practical implications.

The study results indicate that various factors of social media play a role in shaping the public’s attitudes toward environmental protection and subjective norms. People are turning to social media for information on new environmental issues because it is seen as a popular source of information. Consistent with the study’s prediction, SEM results demonstrated positive relationships between social media usage and environmental attitudes and subjective norms (supporting hypotheses 1a and 1b). The result is consistent with Trivedi et al. (2018) and Sujata et al. (2019), who suggested that the use of social media platforms provides feedback loops that promote social benefits, consumption patterns, well-being, and favorable attitudes toward the environment. In addition, Santoso (2021) stated that interactions with different social influences, feedback, and trends on social media can impact how individuals view societal norms and expected behaviors. This notion corresponds with the study’s findings that social media usage has a profound influence on subjective norms which is also in line with the social impact theory that explains the impact of information sources closeness to the individuals due to the increased immediacy and strength of the sources. Online interpersonal influence impacts environmental attitudes and subjective norms, supporting hypotheses 2a and 2b. This aligns with Minhas and Furqan (2023) finding that one’s environmental attitude is shaped by their perception of the environment influenced by information from friends, family, and social circle. Online interpersonal influence shapes environmental attitudes, leading to a positive attitude toward the environment. The three elements of social impact theory (number, immediacy, and strength) helped predict the potential effect of online interpersonal influence as online platforms allow individuals to share their viewpoints and experiences, leading to strong bonds in online social circles due to frequent internet and social media use (Chung and Austria, 2010). According to Bedard and Tolmie (2018), people often develop strong bonds with others in their online social circles as a result of regular internet and social media usage. This argument aligns with the results of the current study.

The study found no significant impact of social networking site involvement on environmental attitude, contradicting hypothesis 3a possibly due to limited content on social media regarding environmental concerns. Additionally, casual interaction with environmental content may not result in meaningful changes in attitudes or actions due to limited comprehension or absorption of information (Meng et al., 2023). Social networking sites involvement was positively related to subjective norms, supporting hypothesis 3b. This accords with Han et al. (2018) study which found that discussing environmental issues on social media can make users conform to subjective norms under social pressure, especially when their peers are involved. Furthermore, hypotheses 4a and 4b, which suggested that there is a direct positive relationship between exposure to social media content and environmental attitude, and subjective norms was validated. This implies that the more people are exposed to different social media content on various platforms, the more likely their attitudes toward environmental protection will be shaped through persuasive content and storytelling. This aligns with the argument made by Hu et al. (2023) that increasing awareness and knowledge through social media platforms exposure is crucial in shaping public attitudes. Similarly, the finding that exposure to social media content influences subjective norms accords with Yang et al. (2023) study which contended that social media platforms frequently show users content that mirrors the popular opinions, trends, and behaviors of their peers which makes them feel compelled to adhere to these standards in order to be accepted by others or avoid being left out. In this regard, governments and affiliated organizations can consistently utilize social media to disseminate information on environmental conservation, improve public awareness of the importance of protecting the environment and their subjective standards for eco-friendly actions, encourage individuals to engage in environmentally conscious behaviors, and encourage sustainable consumption habits in society.

The positive relationship between user generated content, environmental attitude, and subjective norms (H5a and 5b) supports Kim et al. (2017) suggestion that user participation in content generation leads to greater engagement, increasing the likelihood of sustainable behavioral change. The study showed that social media UGC impacts a destination’s image, attitudes, and intentions to visit. Langley and van den Broek (2010) also reported that environment related UGC aims to promote persuasive communication for responsible tourist behavior. Sharing personal experiences and opinions about the environment can deepen connections and influence pro-environmental attitudes and behaviors. Similarly, user-created content can shape perceptions of acceptable behavior within social groups, potentially impacting subjective norms (Ballara, 2023). This aligns with social impact theory as social media sites often show user-generated content from friends, family, celebrities, or influencers, which impacts individuals’ adherence to subjective norms. Social media enables immediate access to vast user-generated content from multiple sources, impacting subjective norms (Luca, 2015).

Furthermore, results indicate a positive relationship between environmental attitude and behavioral intentions, supporting hypothesis 6a and aligning with TPB. The positive relationship between environmental attitude and behavioral intentions is supported by Smith and McSweeney (2007), who found that positive attitudes lead to intentions for altruistic, pro-environmental, and pro-social behaviors. The study found no relationship between subjective norm and behavioral intention, contradicting previous studies (Yadav and Pathak, 2016) that suggested subjective norm influences young consumers’ intentions to purchase environmentally friendly products. Additionally, Dalila et al. (2020) and Shanmugavel and Balakrishnan (2023) found that subjective norms influence the intention toward e-vehicles. However, the relationship may be weak due to biases in self-reports and the desire for socially acceptable responses (Caputo, 2017).

The study showed that EA and SN are both positively related to PEB, supporting hypotheses 7a and 7b. These findings align with the assumptions of the TPB, which suggests that attitudes influence the development of pro-environmental behavior (Liu et al., 2020). The results are also consistent with previous studies on environmental attitude and behavior. For example, Malik and Singhal (2017) found that people with strong environmental attitudes are more likely to buy and go out of their way to purchase eco-friendly products, and their attitudes strongly impact their pro-environmental behavior. Similarly, Bissing‐Olson et al. (2013) found that a positive attitude toward the environment leads to pro-environmental actions by employees. A positive relationship exists between SN and PEB, which aligns with McDonald and Crandall (2015) study indicating that people are influenced by their peers’ acceptable behaviors. When individuals witness their peers partaking in eco-friendly practices like recycling or using public transportation, they tend to adopt these behaviors themselves to conform with social norms. The study also found a positive relationship between BI and PEB, providing support to hypothesis 8. This aligns with previous studies by Minhas and Furqan (2023) which found that higher green purchase intentions lead to green purchase behavior. The finding also conforms with Liu et al. (2020) and Ajzen (1991)’s TPB who discovered that environmental behavioral intentions significantly positively affect pro-environmental behaviors and that behavioral intentions are direct predictor variables of behaviors (Ajzen, 1991). Similarly, Onel (2017) confirmed that behavioral intention is a crucial determinant in explaining the actual eco-friendly purchase behaviors of consumers demonstrating intention being a good predictor of actual behavior.

Furthermore, the study supports hypotheses 9a by confirming that behavioral intentions mediate the relationship between EA and PEB. This aligns with the TPB, which suggests that intentions indirectly predict behavior. BI did not mediate subjective norms’ impact on PEB, which goes against the TPB. Previous research showed that attitude has a stronger influence on intention than subjective norms (Ham et al., 2015). SN can directly influence PEB without being mediated by BI. PBC had a positive moderation effect on the relationship between intentions and PEB, supporting hypothesis 10. This suggests that individuals with high PBC are more motivated to engage in PEB, strengthening the relationship. Conversely, individuals with low PBC are less likely to act on their intentions, weakening the relationship. The finding aligns with previous research by Luszczynska et al. (2011) that showed individuals with higher PBC are more confident in their ability to perform a behavior, increasing the likelihood of translating their intentions into action.

ANN models largely support the findings of SEM, showing that OIP has a greater influence on EA, followed by UGC. This shows the crucial roles of OIP and UGC in influencing EA, resulting in pro-environmental behavior. However, SEM indicates that SNI has no significant impact on EA, whereas ANN suggests that SMU is less important and has a weaker predictive impact on EA. Additionally, the ANN’s model B showed that SMU has the greatest impact on SN, confirming SEM results. This underscores the need for government, environmental activists, and non-governmental organizations to share environmental information via social media and promote its use by the public. The results show that OIP and SME have weaker effects on SN. In model C, the results align with SEM, with EA being the most important predictor of PEB and BI being the least important.

## 6 Implications, limitations, and future research

The study has some theoretical, methodological, and managerial contributions. Methodologically, the study combined SEM and ANN to analyze data, as they are thought to complement each other (Leong et al., 2015). ANN provides further confirmation of the results from the SEM analysis. Additionally, this approach allows for the examination of both linear and complex nonlinear relationships between different factors and outcomes, as well as a more accurate assessment of the impact of each predictor.

Furthermore, the study supports the theoretical understanding of how social media factors impact pro-environmental behavior via environmental attitudes, subjective norms, and behavioral intentions. It also provides empirical evidence regarding the weak connection between subjective norms and intentions in the TPB. The study found no significant relationship between SN and BI, highlighting a weakness in the TPB. Ajzen (1991) suggests that personal factors can directly influence PEB through subjective norms, bypassing the need for BI. The study supports the TPB, showing that exposure to social media leads to intentions for pro-environmental behavior. This is influenced by changes in environmental attitude and subjective norms and moderated by perceived behavioral control. Individuals with high PBC are more likely to engage in PEB than those with low PBC. This research also contributes to the social media literature on environmental attitudes and subjective norms as determinants of pro-environmental behavior. Most previous studies focus on traditional media, so examining the link between social media factors and pro-environmental behavior is a crucial addition to the current literature on media and the environment.

Practically, the study suggests that environmental education, communication, and policy efforts should focus on encouraging eco-friendly behaviors. Social media has become more significant in shaping public attitudes toward environmental concerns, and governments, organizations, and activists should use it to raise awareness and promote eco-friendly behaviors. Personalized content can cultivate positive environmental attitudes and encourage individuals to adopt environmentally responsible behaviors. Sharing information about the benefits of pro-environmental behavior and practical steps for incorporating these behaviors into everyday life can reinforce individuals’ intentions. Watching videos, reading testimonials, and hearing success stories about environmental practices on social media can encourage people to adopt similar behaviors. Furthermore, stakeholders should recognize the importance of subjective norms on pro-environmental behavior as they can assist in creating community programs that utilize social pressure and group encouragement. Social media can also help build and reinforce environmental communities, fostering joint efforts toward a common goal.

Similar to all other studies, the current research is subject to limitations. One of the potential drawbacks of the current study is that it should have concentrated on a particular age group, potentially resulting in some of the hypotheses being deemed insignificant (Sternad Zabukovšek et al., 2019). Thus, it would be beneficial for future studies to concentrate on a specific age range that is most active on different social media platforms or compare the outcomes among different age categories to identify factors contributing to statistically significant results. Moreover, certain variables in the study, like environmental attitude and subjective norms, might have been influenced by social desirability bias, where people provide false or inaccurate information in surveys or self-reports (Paunonen and LeBel, 2012). This bias arises when individuals prioritize giving socially acceptable responses instead of being truthful or precise. Bias can impair research findings, producing irrelevant conclusions. To tackle this, more research could explore alternative data collection methods.

## 7 Conclusion

Overall, the present study has demonstrated the importance of social media factors in enhancing environmental awareness and protection through environmental attitudes, subjective norms, and behavioral intentions. The study achieved its objectives by employing the theory of planned behavior and social impact theory to support its results and arguments, as well as integrating VBSEM and ANN approaches, which complement each other. Contributions have been made, and recommendations have been suggested that governments, non-governmental organizations, and environmental activists should take advantage of social media platforms to raise awareness and promote eco-friendly behaviors among the public since social media has become more significant in shaping individuals’ perceptions toward environmental protection. However, as Ienna et al. (2022) stated, addressing the environmental crisis will likely necessitate ongoing pro-nature attitudes from individuals, rather than just temporary reactions to appeals for action. To effectively make a difference, it is essential to shift the focus of conservation efforts toward changing empathy (Russell and Blackburn, 2017). As different social media factors influence environmental attitudes and subjective norms, which have a great impact on behavioral intentions and pro-environmental behaviors, empathy is induced in the process as individuals interact on social media platforms, thereby affecting their attitude toward the environment and their tendency to act sustainably.

## Data availability statement

The original contributions presented in the study are included in the article/supplementary material, further inquiries can be directed to the corresponding author.

## Ethics statement

Ethical review and approval was not required for the study on human participants in accordance with the local legislation and institutional requirements. Written informed consent from the [patients/ participants OR patients/participants legal guardian/next of kin] was not required to participate in this study in accordance with the national legislation and the institutional requirements.

## Author contributions

C-HL: Conceptualization, Data curation, Formal analysis, Funding acquisition, Investigation, Methodology, Project administration, Resources, Supervision, Validation, Writing – original draft, Writing – review & editing.
